# Special Issue “Multimodality Imaging in Cardiomyopathies”

**DOI:** 10.3390/jcm11144197

**Published:** 2022-07-20

**Authors:** Antonello D’Andrea, Eduardo Bossone, Stefano Palermi

**Affiliations:** 1 Department of Cardiology, Umberto I Hospital, 84014 Nocera Inferiore, Italy; 2 Unit of Cardiology, Department of Translational Medical Sciences, University of Campania “Luigi Vanvitelli”, Monaldi Hospital, 80131 Naples, Italy; 3 Division of Cardiology, Antonio Cardarelli Hospital, 80131 Naples, Italy; ebossone@hotmail.com; 4 Public Health Department, University of Naples Federico II, 80131 Naples, Italy; stefanopalermi8@gmail.com

Multimodality imaging has a crucial role in the identification and management of patients with suspected cardiomyopathies. Indeed, this approach provides structural and functional details that help in stratification and prognosis of these patients., and variables that facilitate risk stratification and prognosis evaluation. History, physical examination, and electrocardiogram (ECG) are always the first moves in evaluating a patient with cardiac disease. Echocardiography, along with its new functions, is the most common second-line test used to diagnose a cardiomyopathy by depicting structural and functional aberrations even if its role in screening procedures is growing [1]. However, echocardiographic have a limited role in identifying the underlying etiology, since these are often non-specific. Therefore, the use of cardiac magnetic resonance (CMR) [2], cardiac computer tomography (CCT) [3] and nuclear scintigraphy [4] is increasing and new evidence is rapidly accumulating.

Therefore, this Special Issue in the *Journal of Clinical Medicine* (*JCM*) collects high-quality scientific contributions about multimodality imaging in cardiomyopathies diagnosis.

The case–control study by Sarnecki et al. [5] had the aim to assess the structure and functions of the left ventricle (LV) and the right ventricle (RV) in children with left-ventricular non-compaction (LVNC), a rare condition that can cause sudden cardiac death. They compared 16 children with LVNC and 16 matched controls though CMR and tissue-tracking analysis. They found that LVNC in pediatric patients is related with LV enlargement (higher end-diastolic volume) and impaired LV systolic function (lower ejection fraction and lower strain parameters). Moreover, children with LVNC had increased RV trabeculations and asymptomatic impairment of RV myocardial strain.

The study by Cavigli et al. [6] explored the effect induced by marathon sports in a population of 68 healthy master amateur athletes in enhancing arrhythmic events. Through a continuously recorded single-lead ECG, an echocardiogram, and a 12-lead ECG before and after the race, the authors denied the hypothesis of an acute atrial dysfunction induced by ultra-endurance exercise. Indeed, even if higher P wave voltages and more ECG criteria for atrial enlargement were found, atrial longitudinal and contraction strains were normal also after the physical effort and exercise-induced supraventricular arrhythmias were uncommon.

The review by Rimbas et al. [7] focused on transthyretin amyloid cardiomyopathy (ATTR-CM), a condition with widespread amyloid extracellular myocardial deposits, which can lead to heart failure, having high treatment and diagnostic costs. Early diagnosis is crucial for a better therapeutic strategy. The authors concluded that a key diagnostic approach is based on the integration of multimodality cardiac imaging studies (speckle-tracking echocardiography, CMR, cardiac scintigraphy, and CCT) and histological, genetic, and hematological assessments (inflammation, oxidative stress, endothelial dysfunction, and disregulated vasculature structure) in a consecutive algorithm.

Monda et al. [8] applied the concept of multimodality imaging in the context of hypertrophic cardiomyopathy (HCM), a dangerous disease featured by an increased LV wall thickness, which is one of the possible reasons for sudden cardiac death. The multimodality approach is crucial both to first confirm the diagnosis and then to start an adequate therapy. While echocardiography is still the first-line diagnostic tool, the role of CMR, CCT, and different cardiac nuclear imaging techniques will increase in the future, given their potential role in explaining further aspect of this pathology, and to uncover some possible secondary form of HCM, such as Fabry disease and cardiac amyloidosis.

Finally, Sperlongano et al. [9] investigated the role of multimodality imaging in athlete’s heart, a range of structural and functional adaptations that occur in the heart of athletes [10], which may overlap with some cardiac diseases. The authors suggested that clinicians adopt a step-by-step approach in order to have an effective diagnostic process, starting with screening tools (history, physical examination, and ECG, with the potential role of standard echocardiography) and ending with more complex techniques (speckle-tracking echocardiography, stress echocardiography, CCT, and CMR).

Several interesting findings come from these articles. The multimodality approach is always a good choice when evaluating complex cardiac diseases, such as HCM [8], ATTR-CM [7], and LVNC [5], and in a sports cardiology context [6,9]. The physicianshould be able to select the right imaging technique for each clinical scenario following a reasoned step-by-step approach. Each technique has its own pros and cons, that need to be taken in consideration. 

As Guest Editors, we give special thanks to the reviewers for their professional comments and to the *JCM* team for their robust support. Finally, we sincerely thank all of the authors for their valuable contributions.

## References

[1] Palermi S., Serio A., Vecchiato M., Sirico F., Gambardella F., Ricci F., Iodice F., Radmilovic J., Russo V., D’Andrea A. (2021). Potential role of an athlete-focused echocardiogram in sports eligibility. World J. Cardiol..

[2] Ricci F., Aung N., Gallina S., Zemrak F., Fung K., Bisaccia G., Paiva J.M., Khanji M.Y., Mantini C., Palermi S. (2020). Cardiovascular magnetic resonance reference values of mitral and tricuspid annular dimensions: The UK Biobank cohort. J. Cardiovasc. Magn. Reson..

[3] Pontone G., Rossi A., Guglielmo M., Dweck M.R., Gaemperli O., Nieman K., Pugliese F., Maurovich-Horvat P., Gimelli A., Cosyns B. (2022). Clinical applications of cardiac computed tomography: A consensus paper of the European Association of Cardiovascular Imaging-part I. Eur. Heart J. Cardiovasc. Imaging.

[4] Gimelli A., Pugliese N.R., Buechel R.R., Coceani M., Clemente A., Kaufmann P.A., Marzullo P. (2022). Myocardial perfusion scintigraphy for risk stratification of patients with coronary artery disease: The AMICO registry. Eur. Heart J. Cardiovasc. Imaging.

[5] Sarnecki J., Paszkowska A., Petryka-Mazurkiewicz J., Kubik A., Feber J., Jurkiewicz E., Ziółkowska L. (2022). Left and Right Ventricular Morphology, Function and Myocardial Deformation in Children with Left Ventricular Non-Compaction Cardiomyopathy: A Case-Control Cardiovascular Magnetic Resonance Study. J. Clin. Med..

[6] Cavigli L., Zorzi A., Spadotto V., Mandoli G.E., Melani A., Fusi C., D’Andrea A., Focardi M., Valente S., Cameli M. (2022). The Acute Effects of an Ultramarathon on Atrial Function and Supraventricular Arrhythmias in Master Athletes. J. Clin. Med..

[7] Rimbas R.C., Balinisteanu A., Magda S.L., Visoiu S.I., Ciobanu A.O., Beganu E., Nicula A.I., Vinereanu D. (2022). New Advanced Imaging Parameters and Biomarkers—A Step Forward in the Diagnosis and Prognosis of TTR Cardiomyopathy. J. Clin. Med..

[8] Monda E., Palmiero G., Lioncino M., Rubino M., Cirillo A., Fusco A., Caiazza M., Verrillo F., Diana G., Mauriello A. (2022). Multimodality Imaging in Cardiomyopathies with Hypertrophic Phenotypes. J. Clin. Med..

[9] D’Andrea A., Sperlongano S., Russo V., D’Ascenzi F., Benfari G., Renon F., Palermi S., Ilardi F., Giallauria F., Limongelli G. (2021). The Role of Multimodality Imaging in Athlete’s Heart Diagnosis: Current Status and Future Directions. J. Clin. Med..

[10] D’Andrea A., Mele D., Palermi S., Rizzo M., Campana M., Giannuario G.D.I., Gimelli A., Khoury G., Moreo A., a nome dell’Area Cardioimaging dell’Associazione Nazionale Medici Cardiologi Ospedalieri (ANMCO) (2020). Le “zone grigie” degli adattamenti cardiovascolari all’esercizio fisico: Come orientarsi nella valutazione ecocardiografica del cuore d’atleta. G. Ital. Cardiol..

